# Artificial intelligence for the prevention and prediction of colorectal neoplasms

**DOI:** 10.1186/s12967-023-04258-5

**Published:** 2023-07-03

**Authors:** Kohjiro Tokutake, Aaron Morelos-Gomez, Ken-ichi Hoshi, Michio Katouda, Syogo Tejima, Morinobu Endo

**Affiliations:** 1 Department of Gastroenterology, Nagano Red Cross Hospital, 5-22-1 Wakasato, Nagano, 380-8582 Japan; 2 Elect Nano, Mesa, AZ USA; 3 Research Initiative for Supra-Materials, Shinshu University, 4-17-1 Wakasato, Nagano, 380-8553 Japan; 4 Department of Health Checkup Center, Nagano Red Cross Hospital, 5-22-1 Wakasato, Nagano, 380-8582 Japan; 5 Research Organization for Information Science & Technology, 2-32-3, Kitashinagawa, Shinagawa-ku, Tokyo, 140-0001 Japan

**Keywords:** Colorectal cancer, Screening, Blood data, Machine learning, Artificial intelligence

## Abstract

**Background:**

Colonoscopy is a useful as a cancer screening test. However, in countries with limited medical resources, there are restrictions on the widespread use of endoscopy. Non-invasive screening methods to determine whether a patient requires a colonoscopy are thus desired. Here, we investigated whether artificial intelligence (AI) can predict colorectal neoplasia.

**Methods:**

We used data from physical exams and blood analyses to determine the incidence of colorectal polyp. However, these features exhibit highly overlapping classes. The use of a kernel density estimator (KDE)-based transformation improved the separability of both classes.

**Results:**

Along with an adequate polyp size threshold, the optimal machine learning (ML) models’ performance provided 0.37 and 0.39 Matthews correlation coefficient (MCC) for the datasets of men and women, respectively. The models exhibit a higher discrimination than fecal occult blood test with 0.047 and 0.074 MCC for men and women, respectively.

**Conclusion:**

The ML model can be chosen according to the desired polyp size discrimination threshold, may suggest further colorectal screening, and possible adenoma size. The KDE feature transformation could serve to score each biomarker and background factors (health lifestyles) to suggest measures to be taken against colorectal adenoma growth. All the information that the AI model provides can lower the workload for healthcare providers and be implemented in health care systems with scarce resources. Furthermore, risk stratification may help us to optimize the efficiency of resources for screening colonoscopy.

**Supplementary Information:**

The online version contains supplementary material available at 10.1186/s12967-023-04258-5.

## Background

Despite evidence that several colorectal cancer (CRC) screening strategies can reduce CRC mortality, screening rates remain low. CRC is one of the most common forms of cancer worldwide, causing the third highest number of cancer deaths [1]. Both the incidence and mortality of CRC have risen rapidly in Asian countries [2]. Strong evidence shows that screening for CRC can improve patients’ survival, and many countries have implemented CRC screening programs [3]. Colonoscopy is considered the most accurate test for the early detection and prevention of CRC. For high-risk patients who have a family history of CRC in first-degree relatives, colonoscopy is the recommended screening tool [4]. It has been demonstrated that the mortality rate decreases due to colonoscopic polypectomy [5].

However, the burden on the health care system is overwhelming in many countries [6]. According to studies conducted in Asia-Pacific countries, the reported prevalence of advanced colorectal neoplasms in the general population ranges from 3 to 12.5%, and thus unnecessary colonoscopies would be performed if a colonoscopy were offered to the entire population as the primary screening modality [7].

Computed tomography colonography is an option for CRC screening in asymptomatic average-risk individuals [8]. However, since the corresponding facilities are limited, the number of screening tests is limited, and there is a challenge to reduce radiation exposure and colonic perforation.

Stool-based CRC screening tests are suitable for screening a large population. Multitarget stool DNA (mt-sDNA) testing, which includes multiple molecular assays combined with a hemoglobin immunoassay is available [9]. However, genetic analysis is more expensive than other stool-based tests. The fecal immunochemical test (FIT) is preferred as the primary screening method because it is less expensive than colonoscopy and genetic analyses. The current practice in Japan uses colonoscopy as a confirmatory test for patients who test positive for fecal occult blood; it is considered as a primary screening tool in population screening programs. Unfortunately, FIT-positive tends to be disregarded, and patients do not receive a colonoscopy. For asymptomatic subjects, the most effective motivation to undergo a CRC screening test is a recommendation from a family physician [10]. A new and validated screening method would be a good motivation to receive colonoscopy for subject with a high risk of polyp. Improving awareness of CRC and promoting the physicians’ role are necessary to increase the screening participation rates.

Stratifying the population by risk offers the potential to improve the efficiency of screening. CRC risk models can serve for pre-screening to suggest further actions if a patient has a high probability for developing CRC. Diverse data types have been used to create CRC risk models: routine data, self-completed questionnaire, non-genetic biomarkers and genetic-biomarkers [11, 12].

Artificial intelligence (AI) is a tool that can be applied in gastroenterology. In particular, AI has been used for the identification of polyps based on colonoscopy images [13, 14]. AI models may also use datasets other than colonoscopy images, such as blood cell counts, urine analysis, gene expression, and other biomarkers [15–18]. Among the diversity of medical data, blood biomarkers have been used for predicting ovarian cancer [19], human aging [20], renal injury [21], breast cancer [22], atrial fibrillation [23] and other disorders. The use of blood biomarkers for predicting colorectal adenoma is thus promising, possibly reducing the risk for CRC if done in a timely manner. In particular, blood biomarkers of inflammation have been used to predict the presence of colorectal adenoma [24].

We investigated whether artificial intelligence could predict colorectal neoplasia, toward the goals of predicting and preventing CRC. In the present study, we developed an AI pipeline to obtain models that identify patients with high likelihood to exhibit colorectal neoplasia, using accumulated data from medical practice. The insights and developed models may serve as a highly efficient guide to prevent and diagnose colorectal adenomas.

## Methods

Our Nagano Red Cross Hospital is located in Nagano Prefecture in Japan. The number of beds in the hospital is 680 beds, and 16,000 patients are hospitalized annually, and is responsible for emergency medical care in the region. In addition, the Health Checkup Center conducts about 4400 health examinations a year. As a primary checkup for colorectal cancer screening, everyone is undergoing FIT. Many patients whose FITs were positive would be recommended to have a colonoscopy at our hospital at a later date. Even if the FIT is negative, sometimes there are opportunities to consult our hospital for some kind of abdominal symptoms and have a colonoscopy. We retrospectively analysed the data of consecutive patients who had undergone a medical checkup and colonoscopy within 1 year after a medical checkup at Nagano Red Cross Hospital in 2015–2020. We extracted physical findings, lifestyle, and blood test data from a medical checkup database, and polyp data from the endoscope database. If the patients received a positive result on the FIT at a medical checkup, they were advised to undergo a colonoscopy. Even the patients with a negative FIT result could undergo a colonoscopy if they desire to receive screening test. Other patients underwent a colonoscopy several months after their medical checkup, for other reasons. For example, some patients were treated due to abdominal symptoms a few months after the medical checkup and underwent a colonoscopy within 1 year.

The inclusion criteria was male or female > 20 years old, those who have undergone both medical checkup and colonoscopy at our hospitals. The subject was confirmed s responsible for emergency medical caretheir physical condition on the morning of the medical checkup day, and had a blood test with asymptomatic status without fever, respiratory symptoms, digestive symptoms, or any subjective symptoms. The time between medical checkup and endoscopy was no more than 365 days. The reasons for undergoing a colonoscopy were not considered. The exclusion criteria included the following: history of colorectal surgery or colorectal cancer, familial adenomatous polyposis, Lynch syndrome, and any contraindication for colonoscopy. Subjects who have received the second colonoscopy within the research period also have been excluded.

### Colonoscopy and colorectal neoplasms

The hospital gastroenterology department has experienced gastroenterologists who perform colonoscopies. All the subjects received same-day bowel preparation. The colonoscopist spent > 6 min for each scope withdrawal. All detailed findings, including neoplastic and non-neoplastic lesions, were recorded in a standard case report form. All the patients for this study had a colonoscopy and the results gave positive or negative for polyp occurrence. Colonoscopists were required to describe the extent of the examination, document cecal visualization, rate the quality of preparation on the Boston Bowel Preparation Scale (BBPS, 0–3 points for each of 3 colon segments), and record the size and location of lesions. The size of polyps was recorded as the largest observation starting from 1 mm.

## Results

21,447 people had received medical checkup during the target period and 1133 (5.3%) people were positive for FIT. 1290 people had an endoscopy within 365 days. In 274 cases, the same person received several endoscopes, so excluded. Eight patient who have had colon surgery, and five IBD were excluded. After the previous considerations, 1003 people were eligible. As shown in Additional file 1: Table S1, 70.2% were FIT positive. Symptoms such as hematochezia were 9%. A small number of cases were pointed out for abnormalities in image tests such as CT and MRI. In addition, Additional file 1: Table S2 shows the background disease of all patients. The mean age of the study subjects is 60.4 years, 611 subjects are men (60.9%) and 392 are women (39.1%). The average time from medical checkup to colonoscopy was 52.9 days in FIT positive patients and 91.6 days in other patients. Evaluation of the quality of colonoscopes, the mean withdrawal time was 555 s. The bowel preparation was evaluated based on BBPS and mean score was 7.54. Overall, there are 13 cases (1.3%) of invasive cancer, 93 cases ≥ 10 mm adenoma (8.0%), 113 cases of ≥ 8 mm (11.3%), 210 cases of ≥ 6 mm (20.9%), and 547 cases of ≥ 1 mm (54.5%) found in this series. Both malignant and benign lesions were treated as polyps, and the maximum diameter among the observed polyps was measured and recorded.

**Fig. 1:**
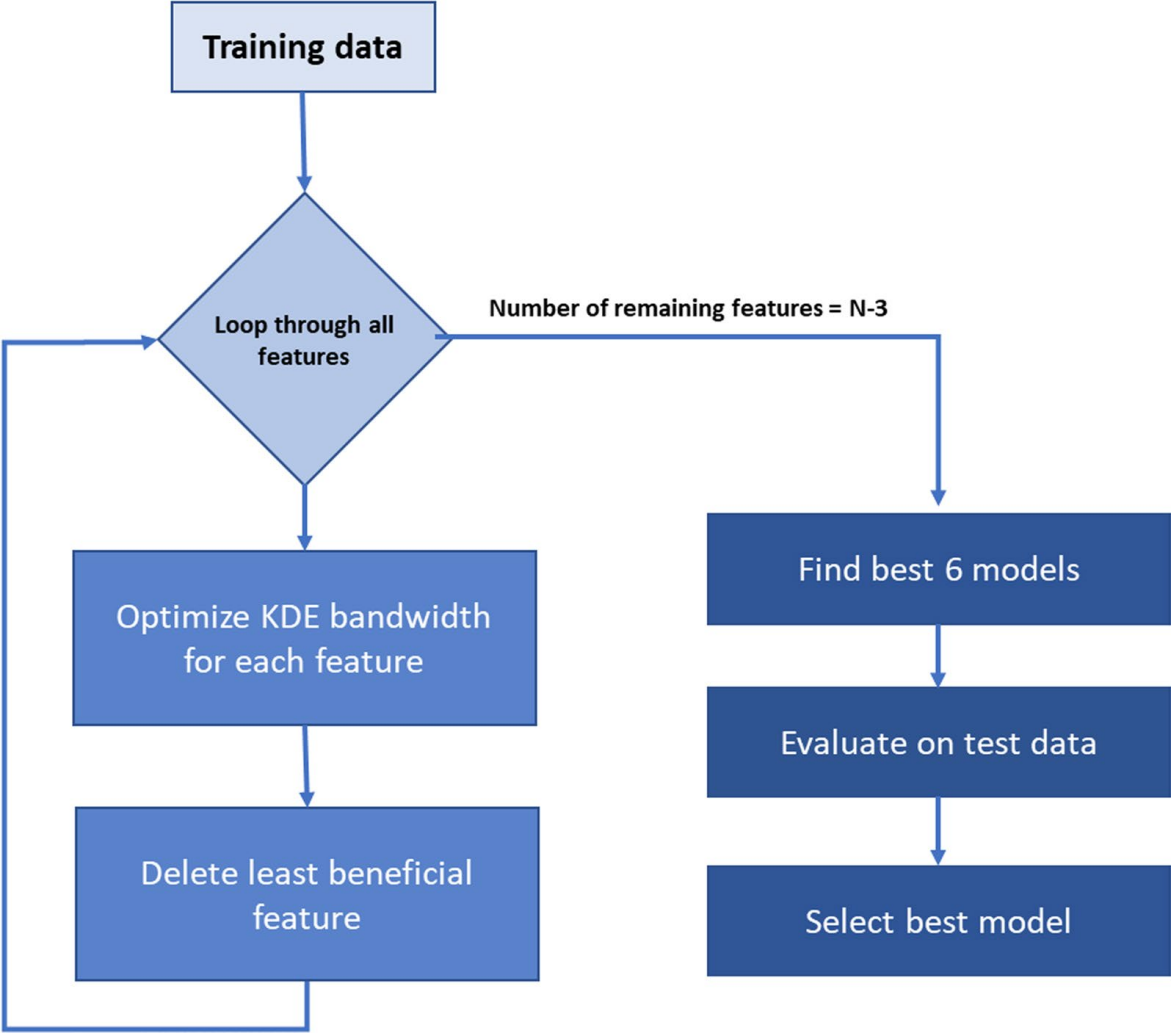
Pipeline to obtain the best optimal after selecting gender, polyp size threshold, and feature transformation. The training data was used for the optimization process. First, the KDE bandwidth was optimized for each feature, then the feature that decreased the most the discrimination performance was eliminated, these steps were iterated until 3 features remained. The best 6 models from all the iterations were selected and evaluated on the test data to select the best of all. All the model selections were based on the MCC value

The overall pipeline consisted of data separation by sex, regrouping patients according to polyp size, transforming the initial data into probabilistic values, optimization of the data transformation parameters, feature selection and selection of the optimal machine learning (ML) method (Fig. 1). This was done to explore a wide variety of models and select the best for the men and the women datasets. A complete description of the method can be found in the supporting information.

The polyp size is widely used to determine clinical importance where the use of a polyp size threshold is commonly assigned between 5 and 10 mm, e.g. in the European society of gastrointestinal endoscopy guidelines for CRC screening [25]. The choice of a polyp size threshold could be due to (1) the risk of developing CRC decreases with polyp size when the polyp(s) are ≤ 10 mm [26], and (2) the human error for detecting polyps increases when the polyp size is smaller [27]. Because of these considerations, the polyp size was used as a threshold; patients with polyp sizes below the threshold are kept in the same group as the patients without polyps (no) and the rest in the group with polyps (yes).

**Fig. 2:**
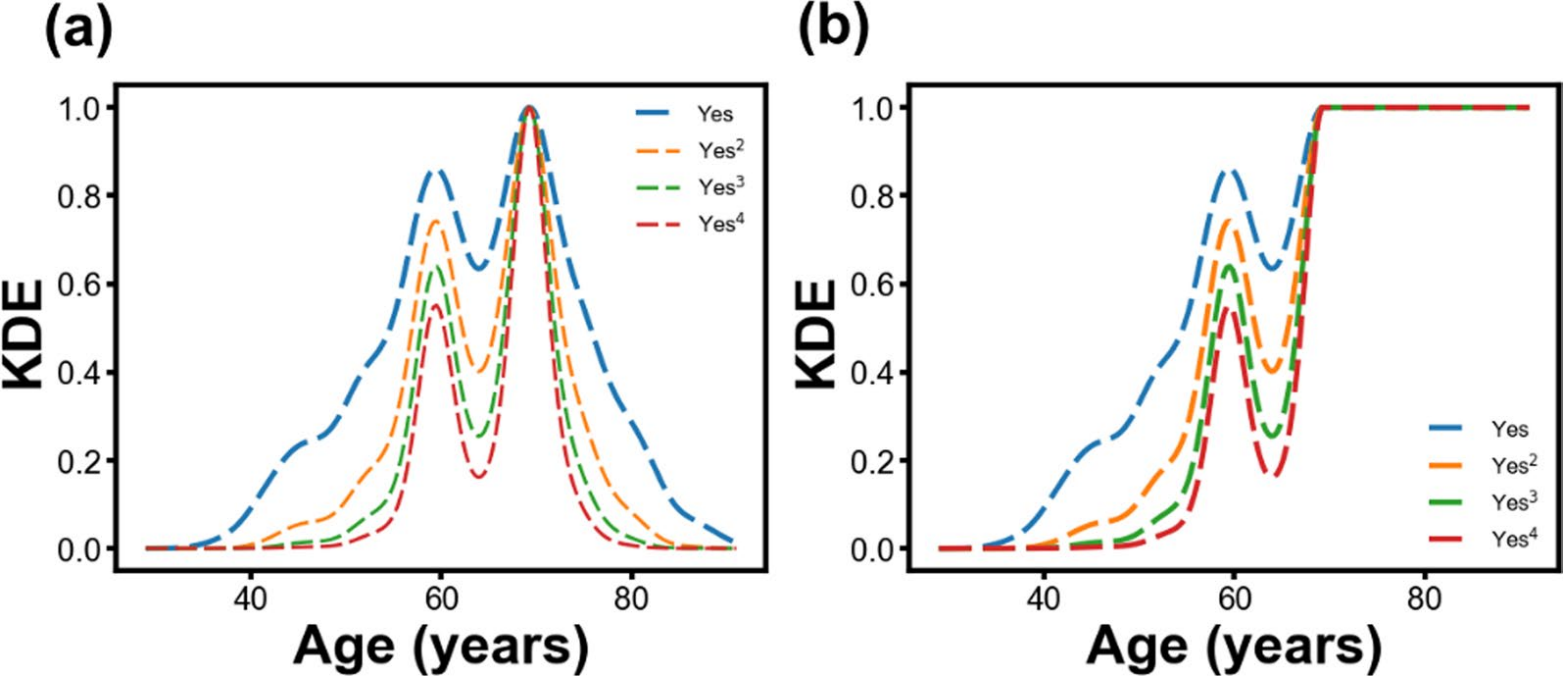
**a** KDE and **b** sigmoid-like KDE functions with different exponents were generated

The input data were treated since the initial numerical features exhibited distributions that are highly overlapping for both incidences of polyps (Additional file 1: Fig. S1). To convert the numerical data into probabilistic values, a KDE-based transformation was implemented. The use of KDE for feature transformation has shown better performance than without KDE-based transformation [28]. Here, for each feature, a KDE was generated using the group of patients with polyps, then the KDE was normalized to 1, and an exponent between 1 and 4 was applied (Fig. 2a).

Sigmoid functions have been widely used to correlate numerical variables with binary classifications, it is used in logistic regression and has been applied in gastroenterology [29, 30]. The KDE was transformed into a sigmoid-like function where the KDE has a value of one before or after the maximum KDE value, according to the Additional file 1: Table S5, and an exponent between 1 and 4 was applied to the sigmoid-like KDE (Fig. 2b).

**Fig. 3:**
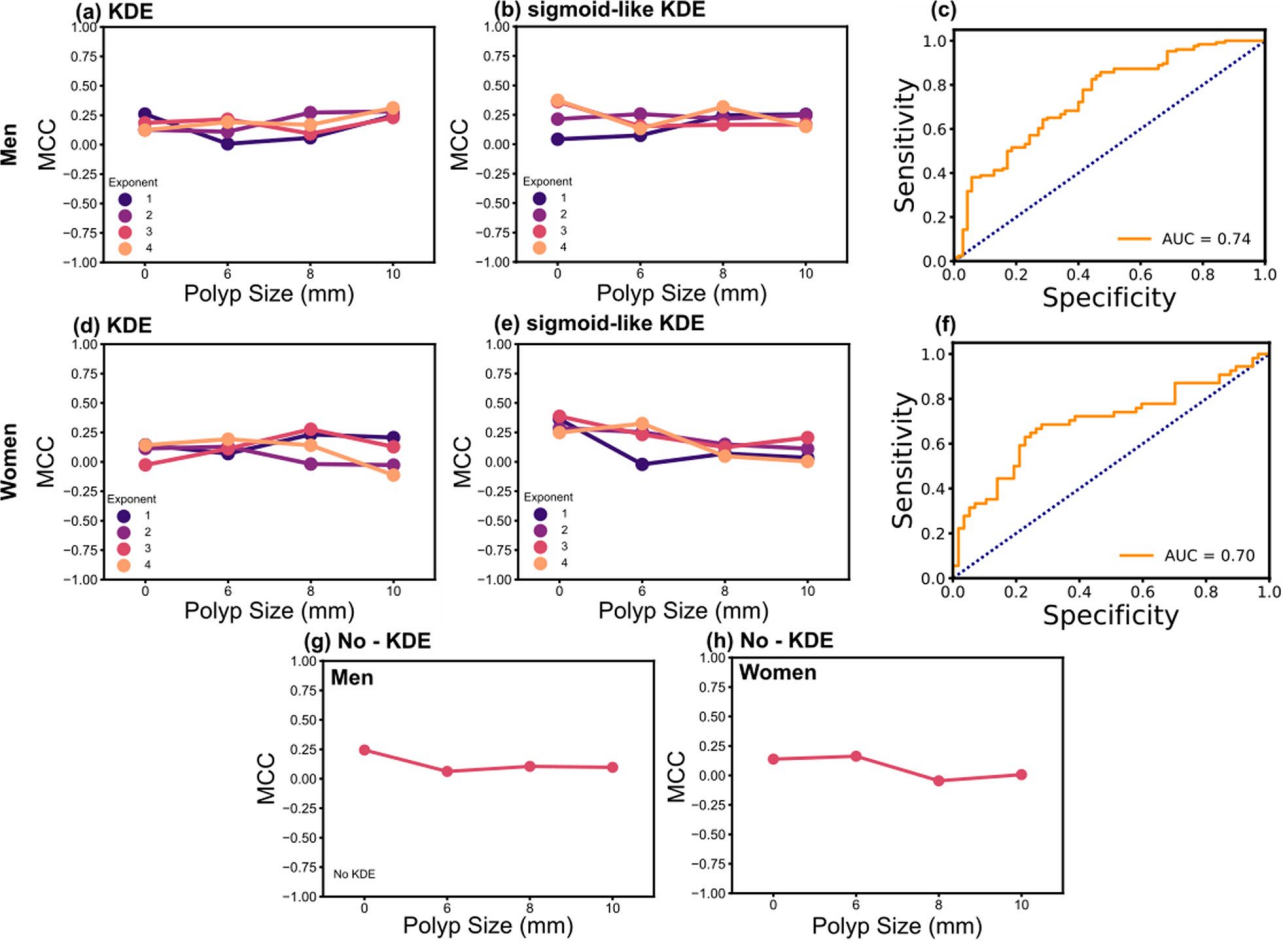
Classification metrics for the **a**–**c** men and **d**–**f** women performed on the test datasets. The Matthews correlation data for **a**, **d** KDE, and **b**, **e** sigmoid-like KDE feature transformations at different polyp size thresholds and KDE exponents are shown. Receiver operating characteristic (ROC) curves on the test data for **c** the men and **f** the women applying the optimal models, the optimal models were those with the highest Matthews correlation: 0 mm polyp size threshold, sigmoid-like KDE-based feature transformation. The Matthews correlation obtained at distinct polyp size threshold without KDE transformation (No - KDE) for the datasets of **g** the men and **h** the women are shown

An optimization algorithm was implemented to obtain the best ML models for men and women (Fig. 3), using a specified polyp size threshold (0, 6, 8, or 10 mm), selection between KDE or sigmoid-like KDE transformations and their respective exponents between 1 and 4. For the present ML models, the sensitivity, specificity, AUC, accuracy, and MCC were calculated with the test data for understanding the performance of the models (Additional file 1: Tables S6–S9, and Fig. 3), and MCC was used as the target metric for the optimization process. For the men’s dataset, the best model is at a polyp threshold size of 0 mm, exponent 4, sigmoid-like KDE-based feature transformation using a Gaussian process classifier, and 0.37 MCC (Fig. 3a–c). For the women’s data, the optimal performance is chosen at 0 mm, exponent 3, sigmoid-like KDE-based feature transformation using A Gaussian process classifier, yielding 0.39 MCC (Fig. 3d–f).

**Fig. 4:**
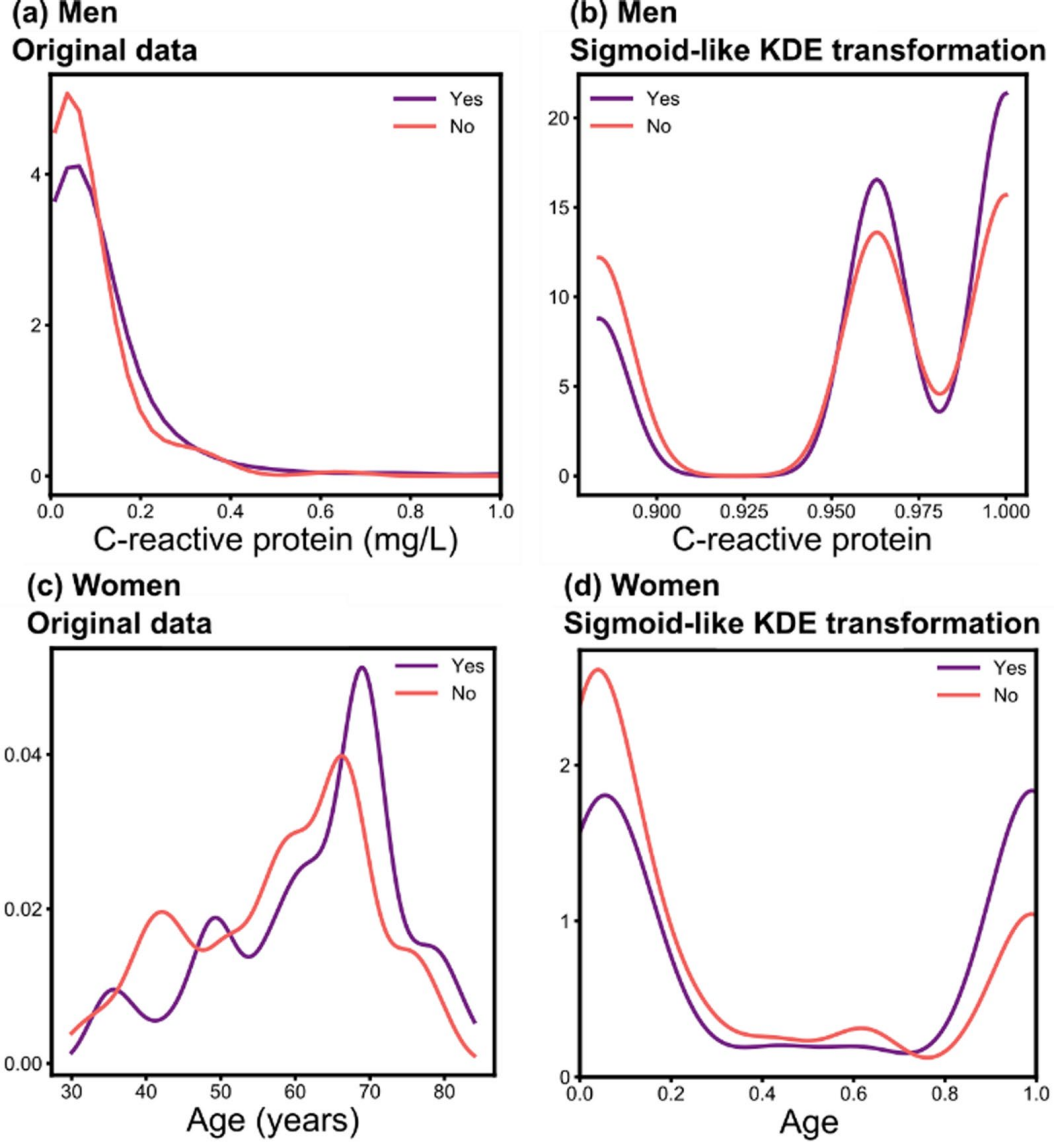
KDE plots of the features with the highest feature importance for **a** men and **c** women and after (**b**, **c**) sigmoid-like KDE transformations

The optimal models with a KDE-based feature transformation have a better discrimination performance than the models without a KDE-based feature transformation considering 0 mm as the polyp size threshold: 0.24 MCC for men, and 0.14 MCC for women (Fig. 3g, h). The higher discrimination performance at 0 mm threshold could be due to the lower probability in patients with smaller polyps to exhibit a false positive given the lack of a previous polyp resection or improvements to their lifestyle. These results demonstrate that the sigmoid-like KDE-based feature transformations enhance the model performance by increasing the separation between the maximum peaks in the feature distributions for each polyp incidence (“yes” and “no”, Fig. 4a–d).

The AI performance metrics are distinct between both sex datasets possibly due to the following reasons. (a) In this study group, the drinking habits and polyp incidence vary among men and women (Additional file 1: Table S10). (b) Additionally, the number of patients with polyps among the women (n = 54) is lower than that among the men (n = 116). (c) The datasets by sex exhibit several features with P-values < 0.05 using the Kruskal-Wallis H-test (Additional file 1: Table S11).

**Fig. 5:**
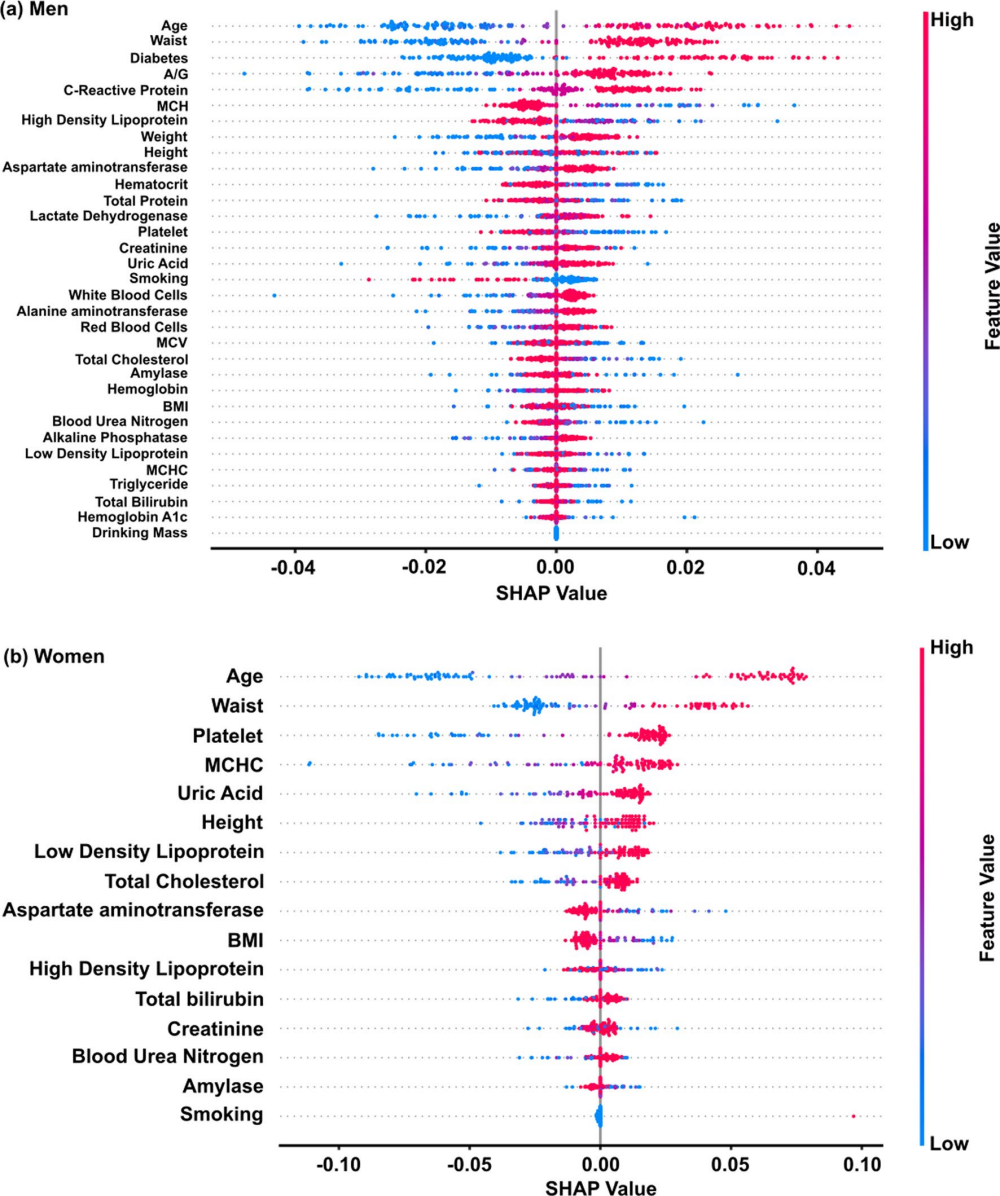
Concerning the feature importance for the **a** men’s and **b** women’s datasets, the shown feature importance were obtained via the SHAP method. The plotted results are those obtained for the optimal models: polyp size threshold 0 mm, sigmoid-like KDE transformation, and KDE exponent 4 for the men; polyp size threshold 0 mm, sigmoid-like KDE transformation and KDE exponent 3 for the women

The optimization algorithm applied on both men and women datasets yielded the selection of 34 and 16 features, respectively, from the initial 39 features. The feature importance was obtained via the Shapley additive explanations (SHAP) method, and for each feature the polyp likelihood can be observed as a higher SHAP value suggests a higher probability to exhibit a polyp (Fig. 5a, b) [31, 32]. For both sexes, the features are mainly related to obesity and alcohol consumption habits, and may be linked to polyp incidence [33–35]. In particular, age and obesity appear as the most important features for both sexes. Features related to obesity exhibit high feature importance, and have been linked with polyp incidence [33, 36]. Both high-density lipoprotein (HDL) and low-density lipoprotein (LDL) are good indicators and they are found within the selected features for each sex, suggesting that lipid profiles can affect the development of colorectal adenomas [37, 38].

**Fig. 6:**
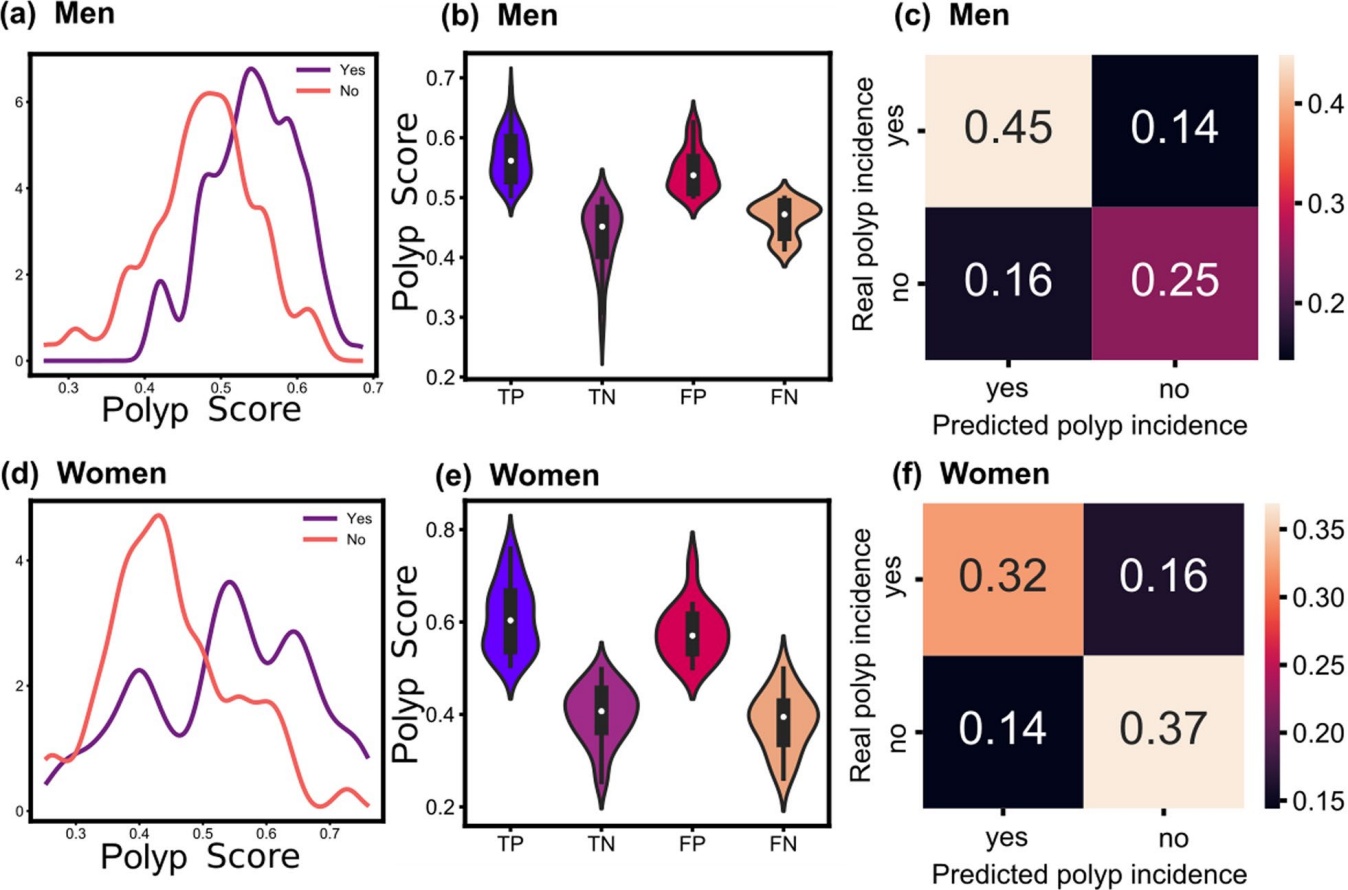
Prediction model performance for the best models for men and women: *0 S-SKDE-4E-GP* and *0 S-SKDE-3E-GP*, respectively. *S* stands for the polyp threshold size, *SKDE* is the sigmoid-like KDE transformation, *E* is the exponent applied on the KDE transformation and *GP* is Gaussian process classifier. **a**, **d** The KDE of the predicted scores for polyp incidence. Each distribution of yes/no is for the true incidence. **b**, **e** Violin plots of the predicted scores for true positive (TP), true negative (TN), false positive (FP), and false negative (FN). **c**, **f** Normalized confusion matrix

AI models can provide a prediction probability or a decision function, here, we consider it as a score value, labelled as polyp score. The KDE of the polyp scores was classified by the real polyp incidence (yes/no), and showed that the optimal model for men exhibits some overlap between both polyp incidence distributions, fortunately, the opposite edges exhibit a lower overlap (Fig. 6a). Surprisingly, the score distributions for true positive (TP), true negative (TN), false positive (FP), and false negative (FN) show that the FN and FP values exhibit a narrower distribution than the TN and TP (Fig. 6b). The confusion matrix shows a higher accurate prediction for “yes” than for the “no” polyp incidence (Fig. 6c).

Regarding the women’s dataset, the KDE polyp scores exhibit a larger gap between the maximum distributions than those for the men (Fig. 6d). In addition, the results reveal similar polyp score distributions among TP-FP and TN-FN (Fig. 6e). From the confusion matrix, the accuracies for the true predictions are higher than the false predictions. In addition, the true predictions exhibit similar accuracies for both polyp incidences (Fig. 6f). The models for the men and women had a low FN, meaning that few patients could be mis-predicted as not having any polyps when a colonoscopy shows a polyp.

## Discussion

People who undergo health checkups are individuals who are concerned about their health and wish to have their health checked. Therefore, the obtained data includes patients that are healthy and those that required a colonoscopy. This provides a dataset to help distinguish between patients who may or may not require a colonoscopy using our AI prediction model. So, using the data of individuals who have undergone health checkups as the subject for AI analysis, specifically for predicting colorectal polyps, is considered to be appropriate. Of course, it is important to be careful of potential data bias or imbalances. The present study includes not only cases for screening for FIT positive, but also patients who have been tested for endoscopy due to abdominal symptoms such as hematochezia or abdominal pain. However, the patients were asymptomatic at the medical checkup. In addition, colonoscopes other than the positive FIT are conducted on average about 90 days later, and it is unlikely that the size and number of polyps will fluctuate during the three months. Although the purpose of colonoscopies is not taken into account, we believe that this study is a useful result as a polyp present or without polyps at the time of the medical checkup.

**Fig. 7:**
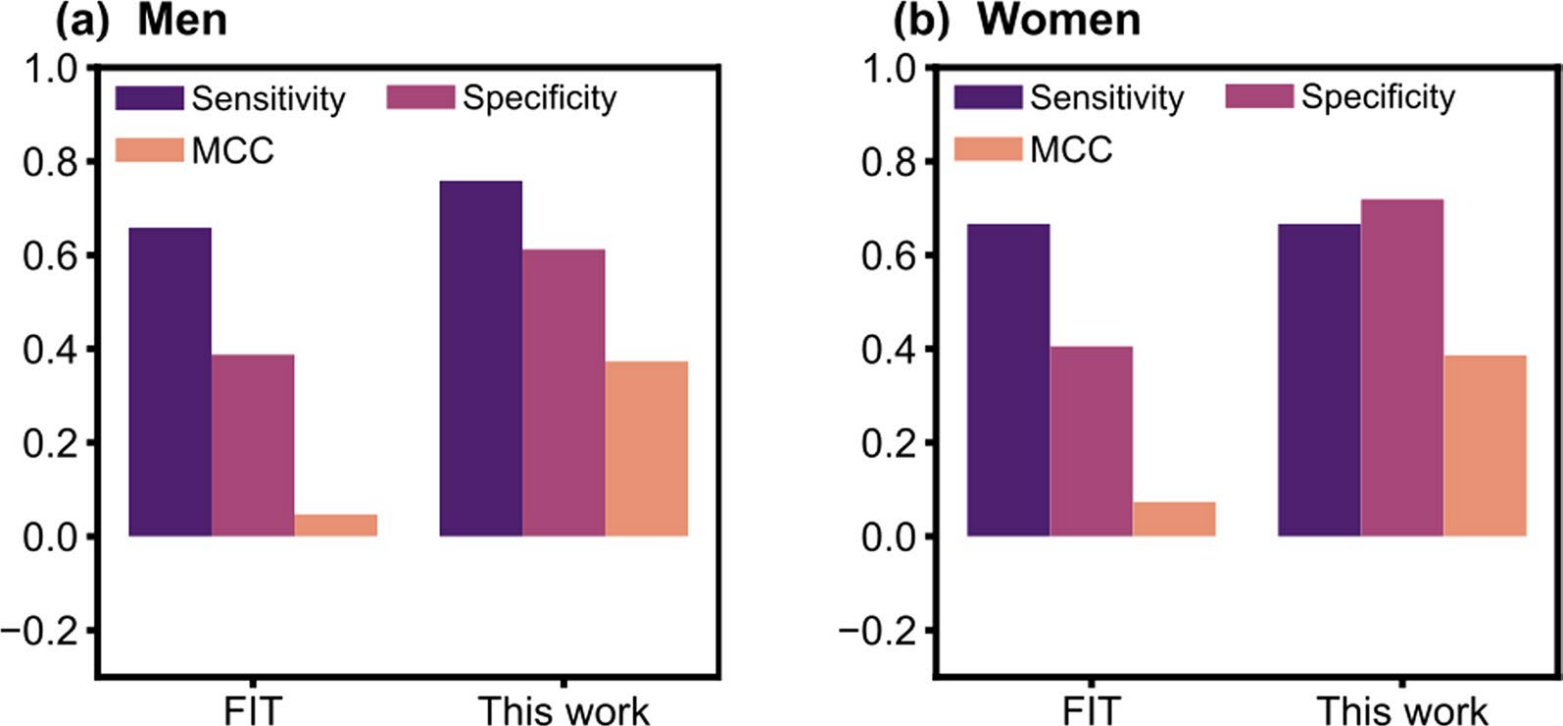
**a**, **b** Sensitivity (*magenta*), specificity (*red*), and Matthews correlation (*orange*). The present models outperform the FIT results for both genders. All the data are presented for the optimal selected models

The FIT is widely used due to its low cost, non-invasive nature, and it has proven efficiency in the reduction of the CRC incidence given its high detection rate of adenomas [39]. The present models outperform the FIT by sensitivity, specificity, and MCC for both sexes (Fig. 7a, b). In addition, the present technique could be better than genetic analysis of stool samples which can identify colorectal adenomas with an average 35% sensitivity (21% standard deviation) [40]. In addition, gene expression analysis can be more expensive and require more specialized equipment than the present blood marker analysis. Therefore, the present models can provide an easier and cheaper screening method.

Unfortunately, some patients convince themselves that the FIT-positive may be caused by hemorrhoidal bleeding, and underestimate the FIT results. Since this model is based on biomarkers and background factors, it could be more persuasive for patients. In addition, the sigmoid-like KDE for each feature could be used as a recommendation for patients to maintain or improve their health lifestyle (e.g., nutrition, alcohol consumption, exercise), and to take care when reaching a particular age. When the KDE transformed values are close to 1 it could be advisable to take measures to avoid the development of colorectal adenoma or carcinoma. The results can be explained from the health check doctor on the day of medical checkup and lead to effective motivation.

The best models outperform the predictions by FIT, however, there could still be room for improvement. Here, the population was considered for a single hospital, with data from other clinics and hospitals it could be possible to obtain better representative data. In addition, clinical history could also help by knowing if a patient is currently taking pharmaceuticals that may reduce polyps such as aspirin. This study includes few cases with a history of colon polypectomy. Some cases which had polyps removed few years ago may be judged to be no polyp, therefore, clinical history could help reduce false negatives. The models may improve with a larger dataset with patients who are subjected to a colonoscopy for the first time.

## Limitations

There are some limitations to the present study. The data for this study were collected in a hospital, where 22 cases with CRC were identified (14 invasive cancers and 8 mucosal cancers), making it difficult to estimate the sensitivity for invasive cancers precisely. We believe that the present models could be enhanced by using a larger dataset of patients who had colonoscopy for the first time as well as medical history such as diabetes, colorectal adenoma, and data from previous colonoscopies.

## Conclusions

The results of the analyses demonstrate that the use of a KDE-based feature transformation can enhance the model performance by increasing the separability among polyp incidence groups. In particular, the sigmoid-like KDE transformation is more beneficial and could provide insights into which biomarker should be monitored to avoid further colorectal adenoma development.

This study demonstrates that risk scoring supported by AI is an effective model to stratify the risk of advanced colorectal neoplasm in asymptomatic subjects undergoing screening tests, which could be further improved with a larger dataset. Each country has its guidelines to determine which treatment or procedure are suggested according to the size of the polyp. The pipeline has the option to select the best model according to a polyp size discrimination threshold. With the given polyp score, the present models could identify patients at a high likelihood of detecting colorectal neoplasm. The procedure is simple enough, requiring only the medical data to be used on the trained models. The models can be used by family physicians and community health care providers to improve screening efficiency, requiring only the input of a physical exam, lifestyle habits, and blood analysis. No additional techniques or costs such as genetic testing are required. We hope that the present findings could aid in the development of AI assisted colorectal adenoma screening.

## Supplementary Information

**Additional file 1: Table S1.** Purpose of colonoscopy. **Table S2.** Past history of all cases. **Table S3.** Statistical description of men data distributions for each feature. **Table S4.** Statistical description of women data distributions for each feature. **Figure S1.** Examples of KDE plots that are (a) less and (b) more alike among patients with (yes) and without (no) polyps, for the men’s dataset. **Table S5.** Direction of highest KDE values. KDE is 1 (representing the highest probability to have polyp) when the feature value is lower or higher than the maximum KDE point. **Table S6.** Optimal model performance for men test data after training on men data at different polyp size threshold and KDE exponent, for KDE transformation (normal) and sigmoid-like KDE transformation (sigmoid). NA stands for not applied, without KDE transformation. The classifiers are adaptive boosting (AdaBoost), Gaussian process, gradient boost, linear discriminant analysis (LDA), linear support vector classifier (Linear SVC), logistic regression, multilayer perceptron (MLP), ridge classifier (Ridge) and support vector classifier (SVC). MCC is the Matthews correlation coefficient. **Table S7.** Optimal model performance for men test data after training at different polyp size threshold without KDE transformation. The classifiers are adaptive boosting (AdaBoost), Gaussian process, gradient boost, linear discriminant analysis (LDA), linear support vector classifier (Linear SVC), logistic regression, multilayer perceptron (MLP), ridge classifier (Ridge) and support vector classifier (SVC). MCC is the Matthews correlation coefficient. **Table S8.** Optimal model performance for women test data at different polyp size threshold and KDE exponent, for KDE transformation (normal) and sigmoid-like transformation (sigmoid). NA stands for not applied, without KDE transformation. The classifiers are adaptive boosting (AdaBoost), Bernoulli Naïve Bayes (BernoulliNB), Gaussian process, linear discriminant analysis (LDA), linear support vector classifier (Linear SVC), logistic regression, multilayer perceptron (MLP), ridge classifier (ridge) and support vector classifier (SVC). MCC is the Matthews correlation coefficient. **Table S9.** Optimal model performance for women test data after training at different polyp size threshold without KDE transformation. The classifiers are adaptive boosting (AdaBoost), Gaussian process, gradient boost, linear discriminant analysis (LDA), linear support vector classifier (Linear SVC), logistic regression, multilayer perceptron (MLP), ridge classifier (Ridge) and support vector classifier (SVC). MCC is the Matthews correlation coefficient. **Table S10.** Percentage of patients with polyps per drinking habit. For drinking frequency, the subgroups were no drinking, sometimes, and everyday. For drinking mass, the subgroups were 3 cans/day. **Table S11.** Kruskal-Wallis H-test results for polyp and gender as independent variables, data is shown for H and P values. **Table S12.** Upper limits to discard data from each feature.

## Data Availability

The Nagano Red Cross Hospital owns the data and is unsuitable for public availability due to hospital restrictions. The code used in this study to develop and evaluate the models are proprietary, therefore, the code is not publicly available.

## Abbreviations

AI: Artificial intelligence
KDE: Kernel density estimator
ML: Machine learning
MCC: Matthews correlation coefficient
CRC: Colorectal cancer
FIT: Fecal immunochemical test
BBPS: Boston Bowel Preparation Scale
SHAP: Shapley additive explanations
HDL: High-density lipoprotein
LDL: Low-density lipoprotein
TP: True positive
TN: True negative
FP: False positive
FN: False negative
