# Supplementary material for: Artificial intelligence for the prevention and prediction of colorectal neoplasms
Source: J Transl Med. 2023 Jul 3;21:431. doi: 10.1186/s12967-023-04258-5 (PMC10318774; doi:10.1186/s12967-023-04258-5)
Supplement: Supplementary file 1 — Additional file 1: Table S1. Purpose of colonoscopy. Table S2. Past history of all cases. Table S3. Statistical description of men data distributions for each feature. Table S4. Statistical description of women data distributions for each feature. Figure S1. Examples of KDE plots that are (a) less and (b) more alike among patients with (yes) and without (no) polyps, for the men’s dataset. Table S5. Direction of highest KDE values. KDE is 1 (representing the highest probability to have polyp) when the feature value is lower or higher than the maximum KDE point. Table S6. Optimal model performance for men test data after training on men data at different polyp size threshold and KDE exponent, for KDE transformation (normal) and sigmoid-like KDE transformation (sigmoid). NA stands for not applied, without KDE transformation. The classifiers are adaptive boosting (AdaBoost), Gaussian process, gradient boost, linear discriminant analysis (LDA), linear support vector classifier (Linear SVC), logistic regression, multilayer perceptron (MLP), ridge classifier (Ridge) and support vector classifier (SVC). MCC is the Matthews correlation coefficient. Table S7. Optimal model performance for men test data after training at different polyp size threshold without KDE transformation. The classifiers are adaptive boosting (AdaBoost), Gaussian process, gradient boost, linear discriminant analysis (LDA), linear support vector classifier (Linear SVC), logistic regression, multilayer perceptron (MLP), ridge classifier (Ridge) and support vector classifier (SVC). MCC is the Matthews correlation coefficient. Table S8. Optimal model performance for women test data at different polyp size threshold and KDE exponent, for KDE transformation (normal) and sigmoid-like transformation (sigmoid). NA stands for not applied, without KDE transformation. The classifiers are adaptive boosting (AdaBoost), Bernoulli Naïve Bayes (BernoulliNB), Gaussian process, linear discriminant analysis (LDA), linear s [file 12967_2023_4258_MOESM1_ESM.docx]

**ADDITIONAL FILE**

**Data treatment**

All the data was treated anonymously to ensure the patients’ privacy. Polyp size was used to create the target data (polyp incidence). In addition, the data of the patients with missing data for height, high-density lipoprotein, alkaline phosphatase, gamma-glutamyl transpeptidase, and total cholesterol were discarded. The data of the patients with data beyond or equal to the outliers shown in Table S8 were also discarded. Categorical features were label encoded into categorical numbers between 0 and 1. The data were divided into two datasets: one for the men and another for the women.

**KDE transformation**

For each feature of the training dataset a kernel density estimation (KDE) was made using the data from the patients with a polyp according to the polyp size threshold. First, the KDE was normalized and then the exponent was applied, varying between 1 and 4. The other transformation function is sigmoid-like, where the KDE is 1 at higher or lower feature values from the maximum KDE according to Table S1. The KDE was used to convert all the original numerical data into values between 0 and 1.

**Machine learning methods search**

Here cross-validation was performed using five-folds. The train dataset was used to create the KDE transformation and was applied on the train and test datasets. Then, all the train and test datasets were normalized according to the minimum and maximum values using to the following equation:

$\frac{x-x_{min}}{x_{max}-x_{min}}$ (1)

The train dataset was balanced according to polyp incidence using the synthetic minority oversampling technique(41) implemented in the imbalanced-learn API.(42) The train and test datasets were used for eleven machine learning methods: gradient boosting classifier, logistic regression, support vector classifier, Adaboost classifier, linear discriminant analysis, Gaussian process classifier, linear support vector classifier, passive-aggressive classifier, ridge classifier, Bernoulli Naive Bayes classifier, and multilayer perceptron classifier. All of these methods were implemented in Python with the Scikit-learn library using the out of the box parameters.(43) For each method the sensitivity, specificity, AUC, accuracy, and Matthews correlation coefficient were calculated.

**ADDITIONAL FILE**

**KDE Optimization and feature selection**

To obtain the highest possible performance, the optimal bandwidth for the KDE transformation of each feature and feature removal was performed by a custom optimization method consisting of a recursive grid search. The target function for optimization was MCC. This was selected to achieve the highest discrimination possible and to avoid bias during prediction.

In the optimization algorithm, all features are initially used, and a KDE bandwidth was given for each feature. Initially, the bandwidth for one feature was varied and the rest remained unmodified, and the machine learning methods search was evaluated. This was repeated for all the features and the KDE bandwidth parameters with the highest target function were selected.

Next, feature selection was performed by stepwise backward feature elimination: removing one feature, keeping the rest and evaluating the performance with the machine learning methods search. The feature with the lowest target function was removed for the entire optimization process.

Afterwards, the KDE bandwidth optimization and feature removal were repeated until three features were remaining.

**ADDITIONAL FILE**

**Table S1.** Purpose of colonoscopy (n=1003).

| **FIT positive** | 704 | (70.2%) |
| --- | --- | --- |
| **Screening(FIT negative)** | 92 | (9.2%) |
| **Symptom** |  |  |
| hematochezia | 29 | (2.9%) |
| constipation | 15 | (1.5%) |
| Other | 46 | (4.6%) |
| **Blood test abnormalities** |  |  |
| elevated tumor marker | 38 | (3.8%) |
| anemia | 4 | (0.4%) |
| **Imaging abnormalities** | 5 | (0.5%) |
| **Other reasons** | 70 | (7.0%) |

**ADDITIONAL FILE**

**Table S2.** Past history of all cases.

| Name of disease | n |  |
| --- | --- | --- |
| Hypertension | 308 | (30.7%) |
| Abnormal lipid metabolism | 212 | (21.1%) |
| Bone fracture | 207 | (20.6%) |
| Appendicitis | 181 | (18.0%) |
| Anemia | 138 | (13.8%) |
| Urinary stone | 120 | (12.0%) |
| Stomach polyp | 115 | (11.5%) |
| Gastric ulcer | 105 | (10.5%) |
| Diabetes | 99 | (9.9%) |
| Duodenal ulcer | 97 | (9.7%) |
| Uterine fibroids | 89 | (8.9%) |
| Pneumonia | 86 | (8.6%) |
| Other heart disease | 78 | (7.8%) |
| Asthma | 72 | (7.2%) |
| Glaucoma | 71 | (7.1%) |
| Thyroid disorder | 69 | (6.9%) |
| Hyperuricemia | 69 | (6.9%) |
| Gallstones | 47 | (4.7%) |
| Hepatitis, liver damage | 44 | (4.4%) |
| Angina | 43 | (4.3%) |
| Nephritis Nephrosis | 40 | (4.0%) |
| Ovarian cyst | 26 | (2.6%) |
| Cerebral infarction | 25 | (2.5%) |
| Chronic bronchitis | 21 | (2.1%) |
| Rheumatoid arthritis | 20 | (2.0%) |
| Myocardial infarction | 20 | (2.0%) |
| Pulmonary tuberculosis | 19 | (1.9%) |
| Cholecystitis | 16 | (1.6%) |
| Uremia, Renal failure | 11 | (1.1%) |
| Cerebral hemorrhage | 8 | (0.8%) |
| Subarachnoid hemorrhage | 4 | (0.4%) |
| Shingles | 3 | (0.3%) |
| Liver cirrhosis | 1 | (0.1%) |

Many cases have multiple diseases

**ADDITIONAL FILE**

**Table S3.** Statistical description of men data distributions for each feature.

| Feature | Mean | 25% | 50% | 75% | Skewness | Kurtosis |
| --- | --- | --- | --- | --- | --- | --- |
| Albumin/Globulin Ratio | 1.539 | 1.39 | 1.54 | 1.68 | 0.216 | 0.834 |
| Age | 62.780 | 56 | 64 | 70 | -0.356 | -0.117 |
| Albumin | 4.313 | 4.1 | 4.3 | 4.5 | -0.148 | 0.118 |
| Alkaline Phosphatase Level | 208.036 | 167.5 | 201 | 239 | 1.454 | 4.524 |
| Alanine Aminotransferase | 24.474 | 16 | 20 | 27 | 8.716 | 123.271 |
| Aspartate Aminotransferase | 25.000 | 19 | 23 | 27 | 16.439 | 328.722 |
| BMI | 23.710 | 21.8 | 23.4 | 25.3 | 0.865 | 1.785 |
| Blood Urea Nitrogen | 15.872 | 12.8 | 15.5 | 18.2 | 2.575 | 22.176 |
| Creatinine | 0.947 | 0.81 | 0.9 | 0.98 | 13.854 | 209.098 |
| C-reative protein | 0.126 | 0.03 | 0.05 | 0.1 | 9.525 | 112.762 |
| Gamma-Glutamyl Transpeptidase | 43.750 | 21 | 30 | 46 | 3.667 | 17.774 |
| Hematocrit | 44.320 | 42 | 44.6 | 46.7 | -0.434 | 0.851 |
| High Density Lipoprotein | 60.797 | 50 | 59 | 70 | 0.935 | 1.666 |
| Height | 169.069 | 165.05 | 169.4 | 173.1 | -0.138 | 0.484 |
| Hemoglobin | 14.709 | 13.9 | 14.8 | 15.6 | -0.618 | 1.198 |
| Lactate Dehydrogenase | 191.795 | 169 | 187 | 209.5 | 1.988 | 11.294 |
| Low Density Lipoprotein | 120.249 | 99 | 119 | 138.5 | 0.530 | 0.597 |
| Systolic Blood Pressure | 130.872 | 120 | 130 | 141 | 0.330 | 0.085 |
| MCH | 31.240 | 30.2 | 31.2 | 32.3 | -0.826 | 5.308 |
| MCHC | 33.182 | 32.6 | 33.2 | 33.8 | -0.457 | 2.378 |
| MCV | 94.208 | 91 | 94 | 97 | -0.154 | 2.270 |
| Diastolic Blood Pressure | 79.730 | 72 | 80 | 87 | 0.451 | 0.848 |
| Platelet | 22.589 | 18.85 | 22.1 | 25.4 | 2.057 | 14.924 |
| Red Blood Cells | 472.140 | 442 | 475 | 501.5 | -0.309 | 0.854 |
| Amylase | 81.367 | 63 | 76 | 96 | 1.383 | 3.737 |
| Total bilirubin | 0.833 | 0.6 | 0.8 | 1 | 1.447 | 3.948 |
| Total Cholesterol | 193.224 | 171 | 191 | 212 | 0.417 | 0.289 |
| Triglyceride | 113.298 | 71 | 98 | 131.5 | 2.979 | 14.633 |
| Total Protein | 7.165 | 6.9 | 7.2 | 7.5 | -0.088 | -0.252 |
| Uric Acid | 6.016 | 5.3 | 6 | 6.8 | 0.147 | 0.236 |
| Waist | 85.562 | 80 | 85 | 90.05 | 0.533 | 1.032 |
| White blood cells | 55.513 | 45.15 | 53.6 | 63.55 | 0.867 | 1.210 |
| Weight | 67.923 | 61.15 | 67 | 73.35 | 0.765 | 1.110 |

**ADDITIONAL FILE**

**Table S4.** Statistical description of women data distributions for each feature.

| Feature | Mean | 25% | 50% | 75% | Skewness | Kurtosis |
| --- | --- | --- | --- | --- | --- | --- |
| Albumin/Globulin Ratio | 1.488 | 1.36 | 1.5 | 1.63 | -0.170 | 1.044 |
| Age | 61.352 | 53.5 | 63 | 70 | -0.524 | -0.354 |
| Albumin | 4.295 | 4.1 | 4.3 | 4.5 | -0.318 | 0.106 |
| Alkaline Phosphatase Level | 209.913 | 166 | 201 | 249.5 | 0.947 | 2.719 |
| Alanine Aminotransferase | 19.268 | 13 | 16 | 21 | 3.372 | 14.748 |
| Aspartate Aminotransferase | 22.728 | 18 | 21 | 25 | 4.861 | 39.917 |
| BMI | 22.236 | 20.05 | 21.8 | 24.25 | 0.755 | 1.551 |
| Blood Urea Nitrogen | 14.576 | 11.9 | 14.2 | 16.5 | 0.744 | 0.589 |
| Creatinine | 0.688 | 0.62 | 0.68 | 0.74 | 0.980 | 2.087 |
| C-reative protein | 0.076 | 0.02 | 0.04 | 0.08 | 5.544 | 39.753 |
| Gamma-Glutamyl Transpeptidase | 24.282 | 14 | 17 | 27 | 4.194 | 25.513 |
| Hematocrit | 40.646 | 38.5 | 40.8 | 42.65 | -0.149 | 0.268 |
| High Density Lipoprotein | 72.868 | 62 | 71 | 82 | 0.827 | 2.033 |
| Height | 155.493 | 151.9 | 155.5 | 159.45 | -0.127 | -0.077 |
| Hemoglobin | 13.220 | 12.6 | 13.3 | 13.9 | -0.500 | 1.244 |
| Lactate Dehydrogenase | 204.638 | 181 | 200 | 221.5 | 1.455 | 3.784 |
| Low Density Lipoprotein | 127.564 | 103.5 | 125 | 146 | 0.509 | 0.440 |
| Systolic Blood Pressure | 125.129 | 112.5 | 125 | 134.5 | 0.404 | 0.191 |
| MCH | 30.131 | 29.3 | 30.4 | 31.35 | -1.820 | 7.288 |
| MCHC | 32.510 | 32.1 | 32.6 | 33.1 | -1.083 | 3.359 |
| MCV | 92.676 | 90 | 93 | 96 | -1.335 | 4.902 |
| Diastolic Blood Pressure | 72.488 | 65 | 72 | 80 | -0.050 | -0.089 |
| Platelet | 23.618 | 19.6 | 23.5 | 26.85 | 0.287 | -0.160 |
| Red Blood Cells | 439.753 | 414 | 439 | 465 | 0.069 | -0.129 |
| Amylase | 81.523 | 62.5 | 79 | 96 | 0.543 | 0.370 |
| Total bilirubin | 0.720 | 0.6 | 0.7 | 0.8 | 1.223 | 2.235 |
| Total Cholesterol | 208.700 | 185 | 206 | 227 | 0.500 | 0.753 |
| Triglyceride | 90.916 | 62.5 | 83 | 111.5 | 1.149 | 1.443 |
| Total Protein | 7.236 | 7 | 7.2 | 7.5 | 0.343 | 3.312 |
| Uric Acid | 4.704 | 4.1 | 4.6 | 5.3 | 0.248 | 1.210 |
| Waist | 80.766 | 74.25 | 81 | 86.8 | 0.205 | 0.422 |
| White blood cells | 49.717 | 40.75 | 48.8 | 55.3 | 1.935 | 7.915 |
| Weight | 53.777 | 48.4 | 53.1 | 58.75 | 0.756 | 2.104 |

**ADDITIONAL FILE**


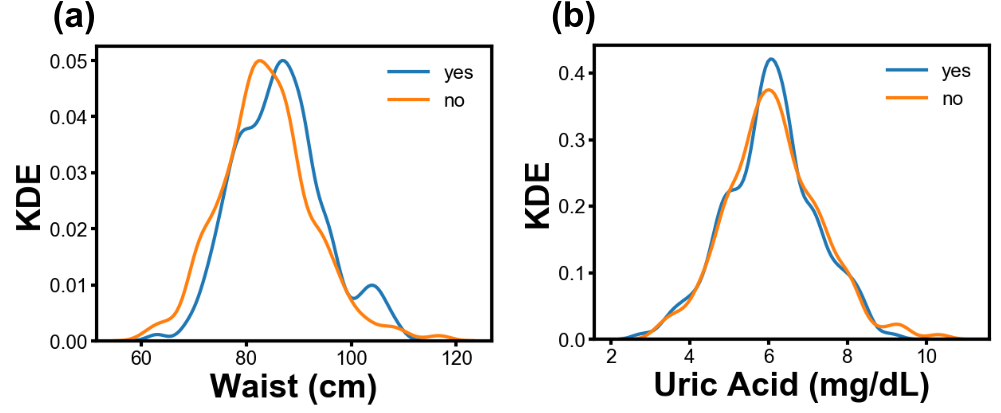


**Figure S1.** Examples of KDE plots that are (a) less and (b) more alike among patients with (yes) and without (no) polyps, for the men's dataset.

**ADDITIONAL FILE**

**Table S5.** Direction of highest KDE values. KDE is 1 (representing the highest probability to have polyp) when the feature value is lower or higher than the maximum KDE point.

| Feature | Men | Women | Feature | Men | Women |
| --- | --- | --- | --- | --- | --- |
| AG_R | lower | lower | MaxBP | higher | higher |
| Age | higher | higher | MCH | lower | lower |
| ALB | lower | lower | MCHC | lower | lower |
| ALP | higher | higher | MCV | lower | lower |
| ALT | higher | higher | MinBP | higher | higher |
| AST | higher | higher | HbA1c | higher | higher |
| BMI | higher | higher | PLT | lower | lower |
| BUN | higher | higher | RBC | lower | lower |
| CRE | higher | higher | S-AMY | higher | higher |
| CRP | higher | higher | T-Bil | higher | higher |
| G-GTP | higher | higher | T-Cho | higher | higher |
| HCT | lower | higher | TG | higher | higher |
| HDL-C | lower | lower | TP | higher | higher |
| Height | higher | lower | UA | higher | higher |
| Hgb | lower | lower | Waist | higher | higher |
| LDH | lower | lower | WBC | higher | higher |
| LDL-C | higher | higher | Weight | higher | higher |

**ADDITIONAL FILE**

**Table S6.** Optimal model performance for men test data after training on men data at different polyp size threshold and KDE exponent, for KDE transformation (normal) and sigmoid-like KDE transformation (sigmoid). NA stands for not applied, without KDE transformation. The classifiers are adaptive boosting (AdaBoost), Gaussian process, gradient boost, linear discriminant analysis (LDA), linear support vector classifier (Linear SVC), logistic regression, multilayer perceptron (MLP), ridge classifier (Ridge) and support vector classifier (SVC). MCC is the Matthews correlation coefficient.

| Polyp  Size | Exponent | Sensitivity | Specificity | AUC | Accuracy | MCC | KDE  Transformation | Classifer |
| --- | --- | --- | --- | --- | --- | --- | --- | --- |
| 0 | 1 | 0.68 | 0.59 | 0.67 | 0.64 | 0.260 | Normal | LDA |
| 0 | 2 | 0.43 | 0.70 | 0.61 | 0.53 | 0.127 | Normal | Gaussian Process |
| 0 | 3 | 0.63 | 0.57 | 0.61 | 0.61 | 0.185 | Normal | MLP |
| 0 | 4 | 0.66 | 0.47 | 0.59 | 0.59 | 0.123 | Normal | MLP |
| 6 | 1 | 0.29 | 0.72 | 0.46 | 0.60 | 0.007 | Normal | LDA |
| 6 | 2 | 0.42 | 0.69 | 0.56 | 0.62 | 0.110 | Normal | SVC |
| 6 | 3 | 0.40 | 0.81 | 0.60 | 0.69 | 0.216 | Normal | SVC |
| 6 | 4 | 0.48 | 0.73 | 0.61 | 0.66 | 0.192 | Normal | Gaussian Process |
| 8 | 1 | 0.33 | 0.74 | 0.52 | 0.67 | 0.058 | Normal | Linear SVC |
| 8 | 2 | 0.48 | 0.83 | 0.71 | 0.78 | 0.273 | Normal | SVC |
| 8 | 3 | 0.47 | 0.64 | 0.62 | 0.61 | 0.093 | Normal | LDA |
| 8 | 4 | 0.19 | 0.93 | 0.62 | 0.80 | 0.169 | Normal | SVC |
| 10 | 1 | 0.33 | 0.91 | 0.69 | 0.83 | 0.247 | Normal | Gradient Boosting |
| 10 | 2 | 0.52 | 0.84 | 0.66 | 0.81 | 0.282 | Normal | SVC |
| 10 | 3 | 0.33 | 0.90 | 0.61 | 0.83 | 0.230 | Normal | SVC |
| 10 | 4 | 0.33 | 0.94 | 0.65 | 0.87 | 0.312 | Normal | SVC |
| 0 | 1 | 0.52 | 0.53 | 0.56 | 0.52 | 0.043 | Sigmoid | LDA |
| 0 | 2 | 0.72 | 0.49 | 0.65 | 0.64 | 0.214 | Sigmoid | SVC |
| 0 | 3 | 0.81 | 0.53 | 0.71 | 0.71 | 0.361 | Sigmoid | Gradient Boosting |
| 0 | 4 | 0.76 | 0.61 | 0.74 | 0.70 | 0.373 | Sigmoid | Gaussian Process |
| 6 | 1 | 0.60 | 0.49 | 0.60 | 0.52 | 0.075 | Sigmoid | Ridge |
| 6 | 2 | 0.63 | 0.65 | 0.68 | 0.65 | 0.257 | Sigmoid | Linear SVC |
| 6 | 3 | 0.59 | 0.59 | 0.61 | 0.59 | 0.154 | Sigmoid | Ridge |
| 6 | 4 | 0.50 | 0.65 | 0.64 | 0.62 | 0.134 | Sigmoid | Logistic Regression |
| 8 | 1 | 0.53 | 0.77 | 0.63 | 0.73 | 0.246 | Sigmoid | SVC |
| 8 | 2 | 0.50 | 0.76 | 0.69 | 0.71 | 0.217 | Sigmoid | SVC |
| 8 | 3 | 0.36 | 0.81 | 0.63 | 0.71 | 0.166 | Sigmoid | SVC |
| 8 | 4 | 0.42 | 0.89 | 0.70 | 0.81 | 0.318 | Sigmoid | SVC |
| 10 | 1 | 0.71 | 0.68 | 0.74 | 0.68 | 0.254 | Sigmoid | MLP |
| 10 | 2 | 0.72 | 0.69 | 0.69 | 0.69 | 0.246 | Sigmoid | Linear SVC |
| 10 | 3 | 0.28 | 0.90 | 0.60 | 0.85 | 0.167 | Sigmoid | Gradient Boosting |
| 10 | 4 | 0.50 | 0.70 | 0.67 | 0.67 | 0.151 | Sigmoid | Gaussian Process |
| 0 | NA | 0.81 | 0.41 | 0.65 | 0.66 | 0.244 | NA | SVC |
| 6 | NA | 0.17 | 0.88 | 0.60 | 0.71 | 0.062 | NA | Ada Boost |
| 8 | NA | 0.97 | 0.12 | 0.54 | 0.26 | 0.105 | NA | Bernoulli NB |
| 10 | NA | 0.25 | 0.86 | 0.59 | 0.80 | 0.097 | NA | Ada Boost |

**ADDITIONAL FILE**

**Table S7.** Optimal model performance for men test data after training at different polyp size threshold without KDE transformation. The classifiers are adaptive boosting (AdaBoost), Gaussian process, gradient boost, linear discriminant analysis (LDA), linear support vector classifier (Linear SVC), logistic regression, multilayer perceptron (MLP), ridge classifier (Ridge) and support vector classifier (SVC). MCC is the Matthews correlation coefficient.

| Polyp Size | Sensitivity | Specificity | AUC | Accuracy | MCC | Classifier |
| --- | --- | --- | --- | --- | --- | --- |
| 0 | 0.347 | 0.804 | 0.529 | 0.507 | 0.158 | Gradient Boosting |
| 0 | 0.537 | 0.549 | 0.564 | 0.541 | 0.082 | Logistic Regression |
| 0 | 0.432 | 0.667 | 0.557 | 0.514 | 0.096 | SVC |
| 0 | 0.253 | 0.725 | 0.501 | 0.418 | -0.024 | AdaBoost |
| 0 | 0.695 | 0.451 | 0.586 | 0.610 | 0.145 | LDA |
| 0 | 0.526 | 0.588 | 0.570 | 0.548 | 0.109 | Gaussian Process |
| 0 | 0.684 | 0.451 | 0.580 | 0.603 | 0.134 | Linear SVC |
| 0 | 0.779 | 0.412 | 0.563 | 0.651 | 0.201 | Passive Aggressive |
| 0 | 0.653 | 0.451 | 0.578 | 0.582 | 0.102 | Ridge |
| 0 | 0.305 | 0.686 | 0.496 | 0.438 | -0.009 | Bernoulli NB |
| 0 | 0.600 | 0.451 | 0.547 | 0.548 | 0.049 | MLP |
| 6 | 0.314 | 0.748 | 0.515 | 0.644 | 0.060 | Gradient Boosting |
| 6 | 0.914 | 0.207 | 0.517 | 0.377 | 0.136 | Logistic Regression |
| 6 | 0.457 | 0.613 | 0.514 | 0.575 | 0.061 | SVC |
| 6 | 0.600 | 0.333 | 0.488 | 0.397 | -0.060 | AdaBoost |
| 6 | 1.000 | 0.036 | 0.549 | 0.267 | 0.094 | LDA |
| 6 | 0.771 | 0.351 | 0.516 | 0.452 | 0.112 | Gaussian Process |
| 6 | 0.943 | 0.117 | 0.531 | 0.315 | 0.084 | Linear SVC |
| 6 | 0.857 | 0.270 | 0.512 | 0.411 | 0.127 | Passive Aggressive |
| 6 | 0.857 | 0.162 | 0.525 | 0.329 | 0.023 | Ridge |
| 6 | 0.771 | 0.261 | 0.494 | 0.384 | 0.032 | Bernoulli NB |
| 6 | 0.714 | 0.342 | 0.506 | 0.432 | 0.051 | MLP |
| 8 | 0.059 | 0.760 | 0.493 | 0.678 | -0.141 | Gradient Boosting |
| 8 | 0.529 | 0.318 | 0.455 | 0.342 | -0.104 | Logistic Regression |
| 8 | 0.294 | 0.736 | 0.501 | 0.685 | 0.022 | SVC |
| 8 | 0.176 | 0.760 | 0.467 | 0.692 | -0.048 | AdaBoost |
| 8 | 0.588 | 0.310 | 0.451 | 0.342 | -0.070 | LDA |
| 8 | 0.529 | 0.395 | 0.440 | 0.411 | -0.049 | Gaussian Process |
| 8 | 0.588 | 0.302 | 0.446 | 0.336 | -0.076 | Linear SVC |
| 8 | 0.529 | 0.372 | 0.434 | 0.390 | -0.065 | Passive Aggressive |
| 8 | 0.588 | 0.310 | 0.451 | 0.342 | -0.070 | Ridge |
| 8 | 1.000 | 0.085 | 0.543 | 0.192 | 0.104 | Bernoulli NB |
| 8 | 0.353 | 0.543 | 0.450 | 0.521 | -0.067 | MLP |
| 10 | 0.000 | 0.902 | 0.558 | 0.822 | -0.098 | Gradient Boosting |
| 10 | 0.308 | 0.481 | 0.433 | 0.466 | -0.120 | Logistic Regression |
| 10 | 0.154 | 0.865 | 0.455 | 0.801 | 0.015 | SVC |
| 10 | 0.231 | 0.789 | 0.608 | 0.740 | 0.014 | AdaBoost |
| 10 | 1.000 | 0.030 | 0.422 | 0.116 | 0.052 | LDA |
| 10 | 0.385 | 0.669 | 0.436 | 0.644 | 0.032 | Gaussian Process |
| 10 | 0.538 | 0.323 | 0.425 | 0.342 | -0.083 | Linear SVC |
| 10 | 0.231 | 0.474 | 0.453 | 0.452 | -0.168 | Passive Aggressive |
| 10 | 0.462 | 0.346 | 0.414 | 0.356 | -0.114 | Ridge |
| 10 | 0.231 | 0.496 | 0.388 | 0.473 | -0.156 | Bernoulli NB |
| 10 | 0.231 | 0.624 | 0.447 | 0.589 | -0.086 | MLP |

**ADDITIONAL FILE**

**Table S8.** Optimal model performance for women test data at different polyp size threshold and KDE exponent, for KDE transformation (normal) and sigmoid-like transformation (sigmoid). NA stands for not applied, without KDE transformation. The classifiers are adaptive boosting (AdaBoost), Bernoulli Naïve Bayes (BernoulliNB), Gaussian process, linear discriminant analysis (LDA), linear support vector classifier (Linear SVC), logistic regression, multilayer perceptron (MLP), ridge classifier (ridge) and support vector classifier (SVC). MCC is the Matthews correlation coefficient.

| Polyp  Size | Exponent | Sensitivity | Specificity | AUC | Accuracy | MCC | KDE  Transformation | Classifier |
| --- | --- | --- | --- | --- | --- | --- | --- | --- |
| 0 | 1 | 0.56 | 0.59 | 0.62 | 0.58 | 0.144 | No | Gaussian Process |
| 0 | 2 | 0.46 | 0.65 | 0.58 | 0.56 | 0.114 | No | Ridge |
| 0 | 3 | 0.32 | 0.66 | 0.48 | 0.51 | -0.026 | No | Bernoulli NB |
| 0 | 4 | 0.38 | 0.75 | 0.65 | 0.57 | 0.142 | No | Gradient Boosting |
| 6 | 1 | 0.44 | 0.68 | 0.51 | 0.66 | 0.070 | No | Bernoulli NB |
| 6 | 2 | 0.13 | 0.96 | 0.57 | 0.84 | 0.129 | No | SVC |
| 6 | 3 | 0.27 | 0.85 | 0.66 | 0.77 | 0.112 | No | SVC |
| 6 | 4 | 0.60 | 0.71 | 0.69 | 0.70 | 0.193 | No | LDA |
| 8 | 1 | 0.27 | 0.94 | 0.52 | 0.87 | 0.233 | No | SVC |
| 8 | 2 | 0.25 | 0.72 | 0.51 | 0.68 | -0.018 | No | Linear SVC |
| 8 | 3 | 0.30 | 0.95 | 0.57 | 0.89 | 0.277 | No | Gradient Boosting |
| 8 | 4 | 0.18 | 0.94 | 0.57 | 0.86 | 0.141 | No | ADA Boost |
| 10 | 1 | 0.57 | 0.79 | 0.60 | 0.77 | 0.207 | No | Passive Aggressive |
| 10 | 2 | 0.09 | 0.88 | 0.67 | 0.80 | -0.027 | No | Linear SVC |
| 10 | 3 | 0.33 | 0.84 | 0.50 | 0.80 | 0.128 | No | MLP |
| 10 | 4 | 0.09 | 0.75 | 0.46 | 0.68 | -0.112 | No | Linear SVC |
| 0 | 1 | 0.64 | 0.72 | 0.74 | 0.68 | 0.362 | Yes | LDA |
| 0 | 2 | 0.59 | 0.69 | 0.66 | 0.65 | 0.286 | Yes | Gradient Boosting |
| 0 | 3 | 0.67 | 0.72 | 0.70 | 0.69 | 0.387 | Yes | Gaussian Process |
| 0 | 4 | 0.56 | 0.69 | 0.70 | 0.62 | 0.248 | Yes | Logistic Regression |
| 6 | 1 | 0.13 | 0.84 | 0.67 | 0.75 | -0.022 | Yes | Gradient Boosting |
| 6 | 2 | 0.75 | 0.65 | 0.74 | 0.66 | 0.252 | Yes | Ridge |
| 6 | 3 | 0.70 | 0.69 | 0.73 | 0.69 | 0.231 | Yes | SVC |
| 6 | 4 | 0.67 | 0.82 | 0.91 | 0.81 | 0.325 | Yes | SVC |
| 8 | 1 | 0.20 | 0.88 | 0.59 | 0.82 | 0.070 | Yes | Gradient Boosting |
| 8 | 2 | 0.33 | 0.86 | 0.58 | 0.82 | 0.149 | Yes | SVC |
| 8 | 3 | 0.33 | 0.81 | 0.59 | 0.75 | 0.123 | Yes | SVC |
| 8 | 4 | 0.10 | 0.94 | 0.50 | 0.86 | 0.048 | Yes | SVC |
| 10 | 1 | 0.13 | 0.91 | 0.70 | 0.86 | 0.034 | Yes | Gradient Boosting |
| 10 | 2 | 0.50 | 0.70 | 0.59 | 0.68 | 0.111 | Yes | Passive Aggressive |
| 10 | 3 | 0.17 | 0.98 | 0.61 | 0.94 | 0.206 | Yes | Gradient Boosting |
| 10 | 4 | 0.17 | 0.84 | 0.60 | 0.80 | 0.003 | Yes | SVC |
| 0 | NA | 0.15 | 0.93 | 0.63 | 0.57 | 0.138 | NA | Passive Aggressive |
| 6 | NA | 0.36 | 0.84 | 0.61 | 0.77 | 0.163 | NA | Gradient Boosting |
| 8 | NA | 0.08 | 0.88 | 0.43 | 0.78 | -0.046 | NA | SVC |
| 10 | NA | 0.13 | 0.88 | 0.60 | 0.83 | 0.007 | NA | Gradient Boosting |

**ADDITIONAL FILE**

**Table S9.** Optimal model performance for women test data after training at different polyp size threshold without KDE transformation. The classifiers are adaptive boosting (AdaBoost), Gaussian process, gradient boost, linear discriminant analysis (LDA), linear support vector classifier (Linear SVC), logistic regression, multilayer perceptron (MLP), ridge classifier (Ridge) and support vector classifier (SVC). MCC is the Matthews correlation coefficient.

| Polyp Size | Sensitivity | Specificity | AUC | Accuracy | MCC | Classifier |
| --- | --- | --- | --- | --- | --- | --- |
| 0 | 0.676 | 0.612 | 0.585 | 0.639 | 0.284 | Gradient Boosting |
| 0 | 0.529 | 0.571 | 0.581 | 0.554 | 0.099 | Logistic Regression |
| 0 | 0.471 | 0.510 | 0.530 | 0.494 | -0.019 | SVC |
| 0 | 0.559 | 0.469 | 0.523 | 0.506 | 0.028 | AdaBoost |
| 0 | 0.765 | 0.306 | 0.522 | 0.494 | 0.078 | LDA |
| 0 | 0.441 | 0.612 | 0.544 | 0.542 | 0.053 | Gaussian Process |
| 0 | 0.529 | 0.612 | 0.562 | 0.578 | 0.140 | Linear SVC |
| 0 | 0.500 | 0.612 | 0.560 | 0.566 | 0.111 | Passive Aggressive |
| 0 | 0.529 | 0.571 | 0.563 | 0.554 | 0.099 | Ridge |
| 0 | 0.529 | 0.633 | 0.591 | 0.590 | 0.161 | Bernoulli NB |
| 0 | 0.471 | 0.612 | 0.559 | 0.554 | 0.082 | MLP |
| 6 | 0.083 | 0.873 | 0.462 | 0.759 | -0.047 | Gradient Boosting |
| 6 | 0.667 | 0.437 | 0.508 | 0.470 | 0.074 | Logistic Regression |
| 6 | 0.333 | 0.718 | 0.556 | 0.663 | 0.040 | SVC |
| 6 | 0.083 | 0.930 | 0.346 | 0.807 | 0.018 | AdaBoost |
| 6 | 0.833 | 0.366 | 0.498 | 0.434 | 0.148 | LDA |
| 6 | 0.500 | 0.479 | 0.529 | 0.482 | -0.015 | Gaussian Process |
| 6 | 0.833 | 0.352 | 0.502 | 0.422 | 0.139 | Linear SVC |
| 6 | 0.667 | 0.352 | 0.496 | 0.398 | 0.014 | Passive Aggressive |
| 6 | 0.750 | 0.408 | 0.501 | 0.458 | 0.114 | Ridge |
| 6 | 0.417 | 0.620 | 0.570 | 0.590 | 0.026 | Bernoulli NB |
| 6 | 0.583 | 0.437 | 0.530 | 0.458 | 0.014 | MLP |
| 8 | 0.429 | 0.816 | 0.502 | 0.783 | 0.168 | Gradient Boosting |
| 8 | 0.286 | 0.539 | 0.440 | 0.518 | -0.098 | Logistic Regression |
| 8 | 0.000 | 0.855 | 0.515 | 0.783 | -0.119 | SVC |
| 8 | 0.143 | 0.816 | 0.524 | 0.759 | -0.030 | AdaBoost |
| 8 | 0.714 | 0.224 | 0.444 | 0.265 | -0.041 | LDA |
| 8 | 0.286 | 0.592 | 0.408 | 0.566 | -0.069 | Gaussian Process |
| 8 | 0.571 | 0.421 | 0.412 | 0.434 | -0.004 | Linear SVC |
| 8 | 0.857 | 0.368 | 0.432 | 0.410 | 0.131 | Passive Aggressive |
| 8 | 0.571 | 0.447 | 0.442 | 0.458 | 0.011 | Ridge |
| 8 | 0.429 | 0.632 | 0.579 | 0.614 | 0.035 | Bernoulli NB |
| 8 | 0.143 | 0.632 | 0.403 | 0.590 | -0.131 | MLP |
| 10 | 0.250 | 0.886 | 0.577 | 0.855 | 0.090 | Gradient Boosting |
| 10 | 0.750 | 0.354 | 0.633 | 0.373 | 0.047 | Logistic Regression |
| 10 | 0.250 | 0.823 | 0.687 | 0.795 | 0.041 | SVC |
| 10 | 0.750 | 0.759 | 0.832 | 0.759 | 0.247 | AdaBoost |
| 10 | 0.750 | 0.329 | 0.646 | 0.349 | 0.036 | LDA |
| 10 | 0.750 | 0.405 | 0.617 | 0.422 | 0.068 | Gaussian Process |
| 10 | 0.750 | 0.354 | 0.639 | 0.373 | 0.047 | Linear SVC |
| 10 | 1.000 | 0.291 | 0.643 | 0.325 | 0.139 | Passive Aggressive |
| 10 | 0.750 | 0.367 | 0.633 | 0.386 | 0.052 | Ridge |
| 10 | 1.000 | 0.241 | 0.620 | 0.277 | 0.123 | Bernoulli NB |
| 10 | 0.500 | 0.570 | 0.601 | 0.566 | 0.030 | MLP. |

**ADDITIONAL FILE**

The percentage of patients with polyps for each level of alcohol consumption is found in Table S4. The changes from no alcohol consumption to everyday alcohol consumption is 57% to 66% and 37% to 45% for the men and women datasets, respectively. For the drinking mass, from < 1 serving/day (1 serving = 320 ml) to more than three servings/day the variations are 55% to 77% and 39% to 75% for the men and women datasets, respectively. In this study group, the drinking mass exhibits a higher variance in polyp incidence than drinking frequency, especially in the women dataset.

**Table S10.** Percentage of patients with polyps per drinking habit. For drinking frequency, the subgroups were no drinking, sometimes, and everyday. For drinking mass, the subgroups were < 1 can/day, 1-2 cans/day, 2-3 cans/day and > 3 cans/day.

|  | Men | Women |
| --- | --- | --- |
| Drinking Frequency |  |  |
| No drinking | 57% | 37% |
| Sometimes | 58% | 43% |
| Everyday | 66% | 45% |
|  |  |  |
| Drinking Mass |  |  |
| < 1 can/day | 55% | 39% |
| 1-2 cans/day | 65% | 33% |
| 2-3 cans/day | 63% | 67% |
| > 3 cans/day | 77% | 75% |

**ADDITIONAL FILE**

**Table S11.** Kruskal-Wallis H-test results for polyp and gender as independent variables, data is shown for H and P values.

|  | Gender | Gender |
| --- | --- | --- |
| Feature | **H** | **P** |
| Height | 4.34E+02 | 2.16E-96 |
| Creatinine | 3.02E+02 | 9.41E-68 |
| Weight | 2.84E+02 | 1.04E-63 |
| Uric Acid | 2.45E+02 | 3.31E-55 |
| Hemoglobin | 1.83E+02 | 1.02E-41 |
| Hematocrit | 1.44E+02 | 4.12E-33 |
| High Density Lipoprotein | 1.07E+02 | 4.84E-25 |
| Gamma-glutamyl transpeptidase | 9.37E+01 | 3.65E-22 |
| Mean corpuscular hemoglobin concentration | 8.61E+01 | 1.72E-20 |
| Red blood cells | 8.38E+01 | 5.46E-20 |
| Diastolic blood pressure | 5.99E+01 | 1.01E-14 |
| Mean corpuscular hemoglobin | 5.74E+01 | 3.61E-14 |
| Waist | 4.69E+01 | 7.52E-12 |
| BMI | 4.64E+01 | 9.56E-12 |
| Total cholesterol | 3.54E+01 | 2.70E-09 |
| Total bilirubin | 3.48E+01 | 3.58E-09 |
| White blood cells | 3.48E+01 | 3.58E-09 |
| Alanine aminotransferase | 3.40E+01 | 5.63E-09 |
| Systolic blood pressure | 3.28E+01 | 1.01E-08 |
| Triglyceride | 2.91E+01 | 6.71E-08 |
| Lactate dehydrogenase | 1.78E+01 | 2.45E-05 |
| Blood Urea Nitrogen | 1.44E+01 | 1.46E-04 |
| C-reative protein | 1.13E+01 | 7.83E-04 |
| Mean corpuscular volume | 1.02E+01 | 1.42E-03 |
| Low Density Lipoprotein | 1.01E+01 | 1.49E-03 |
| Platelet | 9.07E+00 | 2.60E-03 |
| Age | 8.12E+00 | 4.37E-03 |
| Aspartate aminotransferase | 7.96E+00 | 4.78E-03 |
| Carcinoembryonic antigen | 3.14E+00 | 7.63E-02 |
| Amylase | 2.39E+00 | 1.22E-01 |
| Total protein | 1.69E+00 | 1.94E-01 |
| Total Protein and Albumin/Globulin | 1.64E+00 | 2.00E-01 |
| Alkaline Phosphatase Level | 4.57E-01 | 4.99E-01 |
| Hemoglobin A1c | 1.92E-01 | 6.61E-01 |
| Albumin | 2.37E-02 | 8.78E-01 |

**ADDITIONAL FILE**

**Table S12.** Upper limits to discard data from each feature.

| Feature | Limit | Units |
| --- | --- | --- |
| Alanine aminotransferase | 110 | units/L |
| Aspartate aminotransferase | 150 | microkat/L |
| Blood Urea Nitrogen | 40 | mg/dL |
| Creatinine | 4 | mg/dL |
| C-reactive protein | 4 | mg/L |
| Platelet | 50 | 10^9^/mL |
| Triglyceride | 600 | mmol/L |
|  |  |  |
